# Analysis of the implementation of the collaboration programme between the headache unit and the liaison psychiatry programme

**DOI:** 10.1192/j.eurpsy.2023.1619

**Published:** 2023-07-19

**Authors:** F. Garcia Lazaro, F. Gotor Sanchez Luengo, E. Garcia Ligero del Rincon, A. Luque Budia

**Affiliations:** Hospital Universitario Virgen del Rocio Sevilla, SEVILLA, Spain

## Abstract

**Introduction:**

Headache is associated with a wide spectrum of comorbid, statistically and biologically related pathological processes. A person with headache is more likely to have a psychological disorder than the rest of the general population, even more so if the headache is chronic. Psychiatric comorbidity has been shown to act as a risk factor in the chronification of headache and may contribute to increased disability. Anxiety and mood disorders are the psychiatric comorbidities that most influence aspects of the disease such as prognosis, response to treatment and even quality of life.

**Objectives:**

To analyse the results of the implementation of a joint consultation between the headache unit and the liaison psychiatry programme.

To evaluate the efficacy of interdisciplinary intervention in patients diagnosed with resistant headache.

**Methods:**

We performed a descriptive analysis of the database of patients included in the headache programme including data on neurological diagnosis, psychiatric diagnosis, type of intervention, referral to psychiatric consultation and number of subsequent revisions.

**Results:**

Diagnoses related to anxious and depressive symptomatology are the most common diagnoses in patients diagnosed with treatment-resistant headache.

In most of the patients analysed in the database a single joint intervention was necessary.

Referral to mental health consultations from the programme did not lead to an increase in urgent demands with a clinical correlation in terms of diagnostic orientation

**Conclusions:**

Joint intervention in the management of these patients has been found to be beneficial in the reinterpretation of symptoms and progressive desensitisation to fear of chronic illness.

Training in symptom detection at the psychopathological level is important for professionals from other areas as well as training in interviewing skills.

More studies are needed to analyse the outcome of joint interventions in patients with difficult-to-manage chronic diseases and their comorbidities.

**Disclosure of Interest:**

None Declared

